# Leiomyoma of the Round Ligament of the Uterus Mimicking Inguinal Hernia

**DOI:** 10.1155/2018/6702494

**Published:** 2018-03-01

**Authors:** Ioulia Maria Christodoulou, Argiris Angelopoulos, Petros Siaperas, Argyrios Ioannidis, Andreas Skarpas, Antreas Tellos, Georgios Velimezis, Ioannis Karanikas

**Affiliations:** ^1^Second Surgical Department, Sismanoglion General Hospital, Marousi, Greece; ^2^Department of Anatomy, Medical School, National and Kapodistrian University of Athens, Athens, Greece

## Abstract

Masses of the round ligament of the uterus are uncommon. Leiomyomas are the most common of them, followed by mesothelial cysts and in some cases endometriosis. The exact incidence is not known, but most cases are frequently encountered during the fertility age. Leiomyomas are benign tumors, which can present as hernias, lymph nodes, or other inguinal masses. Surgical excision is the appropriate treatment. We are presenting a case of a 47-year-old female patient who was admitted to the hospital with a left inguinal mass. Our patient underwent surgery, and a leiomyoma of the round ligament was found.

## 1. Introduction

Leiomyomas are the most frequent tumors of the round ligament of the uterus. In most cases, they present as inguinal masses in women of reproductive age. They can be mistaken for hernias or lymph nodes. Except from inguinal locations, abdominal and vulvar locations have been reported [1, 2]. After surgical excision, the histopathological examination of the specimen provides the final diagnosis.

## 2. Case Presentation

**Figure 1:**
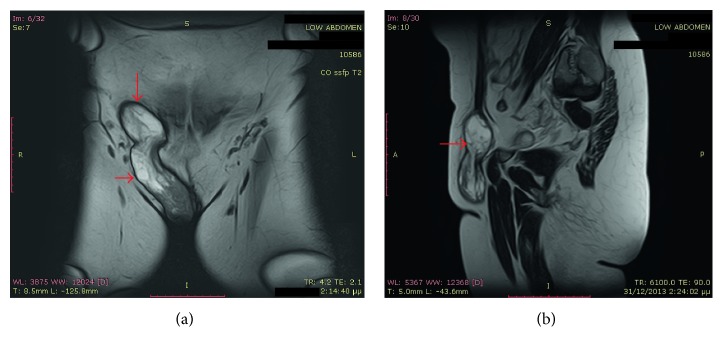
MRI T2W coronal and sagittal images showing a mass of the right round ligament of the uterus (red arrows).

**Figure 2:**
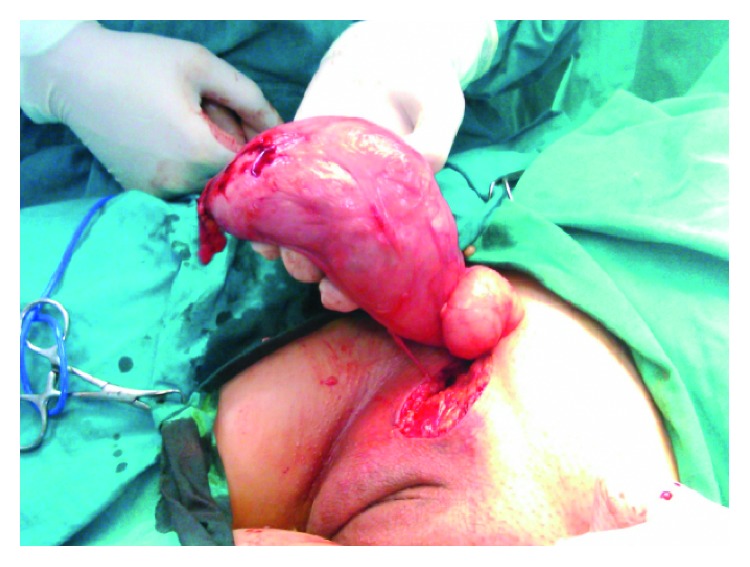
Intraoperative finding of the leiomyoma of the round ligament of the uterus.

**Figure 3:**
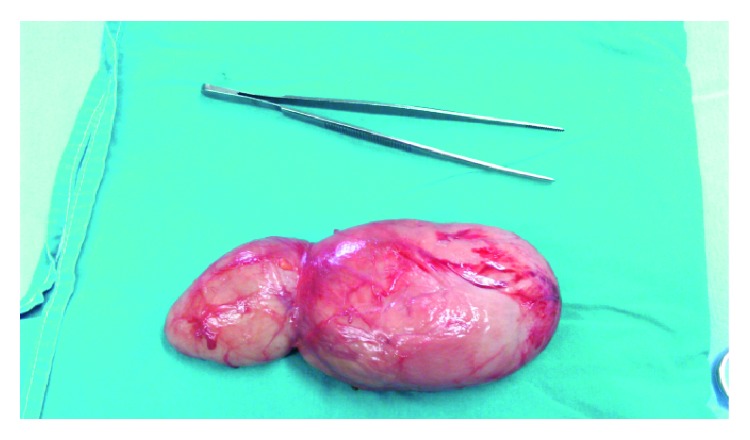
The specimen after the complete surgical excision.

**Figure 4:**
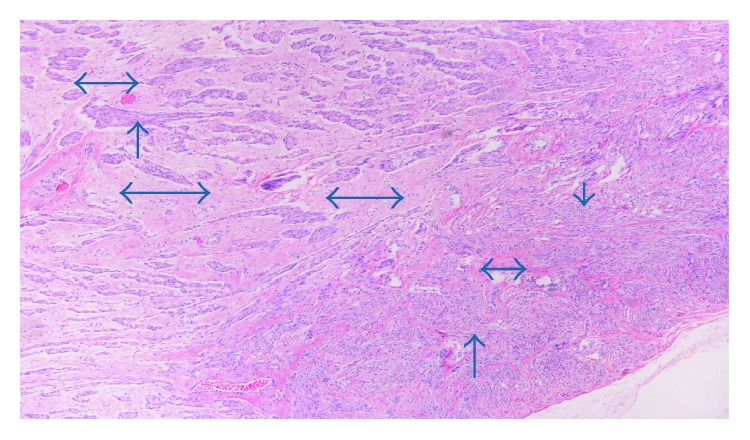
The histopathological examination of the specimen showing intersecting fascicles of smooth muscle cells (simple arrows) separated by vascularized connective tissue (double-headed arrows), confirming the diagnosis of leiomyoma.

A 47-year-old female patient without any significant medical history and a body mass index (BMI) of 26 kg/m^2^ presented with a huge painless mass in the left inguinal region, complaining of sensation of pressure and discomfort in the area. No other symptoms were mentioned. Initially, an inguinal hernia was suspected; however, magnetic resonance imaging (MRI) was recommended. MRI revealed a soft tissue mass of 19 × 6 × 2.5 centimeters (cm), without any pathological lymph nodes. The mass is clearly visible in both coronal and sagittal T2-weighted images. (Figure 1). The patient underwent elective surgery under general anesthesia. After a right inguinal incision, a mass of the right round ligament of the uterus was discovered with no concurrent hernia. The mass was completely excised (Figures 2 and 3). The patient was discharged the next day. No complications were reported in six months of follow-up. Histopathological examination revealed a leiomyoma of the round ligament of the uterus (Figure 4).

## 3. Discussion

Various malignant and benign masses can be found in the inguinal canal. They can affect patients of both genders from 13 to 70 years of age [1]. Fifty percent of round ligament leiomyomas present with uterine fibroids. They most commonly arise from the extraperitoneal end of the round ligament. They are more common on the right side [3, 4]. In the female gender, the round ligament extends from the uterus through the inguinal canal and terminates in the region of the mons pubis and labia majora. Embryologically, it is the female equivalent of the gubernaculum testis. The round ligament of the uterus is responsible for the descent of the ovary from the posterior abdominal wall to the uterus. It is mainly composed of connective tissue, smooth muscle fibers, vessels, and nerves with a mesothelial coating [2, 5].

The most frequently found tumors of the round ligament are leiomyomas. Over 50% of leiomyomas are found in the extraperitoneal portion of the round ligament and most commonly occur in the right side for unknown reasons [5, 6]. Usually, they are discovered in women during the fertility age, but there are also cases which are diagnosed in the postmenopausal period [7]. Their growth is mainly promoted by estrogens. They can mimic inguinal lymphadenopathy or nonreducible/incarcerated inguinal hernia. The differentiation between benign and malignant tumors, especially sarcomas, can be difficult and is possible only after histopathological examination. The major criteria for malignancy are mitotic figures, nuclear atypia, and necrosis [8, 9]. Surgical excision is the treatment of choice which could help the differentiate diagnosis between leiomyoma, inguinal lymphadenopathy, hernia, and a malignant tumor. Imaging techniques before operation, such as computed-assisted tomography (CAT) or MRI, can be helpful but are not always performed prior to surgery [10].

## 4. Conclusion

Leiomyomas of the round ligament can be a possible but rare etiology of the inguinal mass and can be mistaken for an inguinal hernia or lymphadenopathy. Firstly, MRI and, if not possible, a CAT scan can assist when there is diagnostic dilemma, but eventually, surgical exploration provides therapy and defines the exact nature of the mass.
